# *Moringa oleifera* Lam. in Diabetes Mellitus: A Systematic Review and Meta-Analysis

**DOI:** 10.3390/molecules26123513

**Published:** 2021-06-09

**Authors:** Shihori Watanabe, Hiyori Okoshi, Shizuko Yamabe, Masako Shimada

**Affiliations:** 1 Graduate School of Nutritional Science, Sagami Women’s University, 2-1-1 Bunkyo, Minami-ku, Sagamihara, Kanagawa 252-0383, Japan; s2071302@st.sagami-wu.ac.jp (S.W.); s2071301@st.sagami-wu.ac.jp (S.Y.); 2 Department of Nutritional Science, Sagami Women’s University, 2-1-1 Bunkyo, Minami-ku, Sagamihara, Kanagawa 252-0383, Japan; hiyoko.pipi@icloud.com

**Keywords:** *Moringa oleifera*, diabetic rodent models, blood glucose levels, dyslipidemia, meta-analysis

## Abstract

Plant-derived phytochemicals have been interested in as nutraceuticals for preventing the onset and progress of diabetes mellitus and its serious complications in recent years. *Moringa oleifera* Lam. is used in vegetables and in herbal medicine for its health-promoting properties against various diseases including diabetes mellitus. This study aimed to examine an effect of *Moringa oleifera* on diabetic hyperglycemia and dyslipidemia by meta-analyzing the current evidence of diabetic rodent models. Peer-reviewed studies written in English from two databases, PubMed and Embase, were searched to 30 April 2021. Studies reporting blood glucose or lipid levels in diabetic rodents with and without receiving extracts of *Moringa oleifera* were included. Forty-four studies enrolling 349 diabetic rodents treated with extracts of *Moringa oleifera* and 350 diabetic controls reported blood glucose levels. The pooled effect size was −3.92 (95% CI: −4.65 to −3.19) with a substantial heterogeneity. This effect was likely to be, at least in part, modified by the type of diabetic models. Moreover, diabetic hypertriglyceridemia and hypercholesterolemia were also significantly improved in diabetic rodent models treated with *Moringa oleifera*.

## 1. Introduction

The global estimate of diabetes prevalence in the 20–79 year age group was 463 million in 2019 and this figure is expected to reach 700 million by the year 2045 [1]. Type 2 diabetes mellitus (T2DM) is a chronic metabolic disorder that makes up for approximately 90% of DM cases. T2DM is characterized by high blood glucose levels, insulin resistance in the muscle, liver, and adipose tissues, and relative deficiency of insulin secretion from the pancreas. Moreover, patients with DM often develop serious complications including dyslipidemia and cardiovascular diseases, which are the major causes of their increased morbidity and mortality [1]. Therefore, it remains critical to explore new preventative and therapeutic strategies against DM. 

It has been a growing interest whether some natural products including tropical/subtropical plants can be used to prevent and treat patients with DM and its associated complications because of their natural origin and thus relatively limited adverse effects compared with pharmaceuticals [2]. *Moringa oleifera* Lam. (MO), commonly referred to as “drumstick tree,” “horseradish tree,” or “miracle tree”, belongs to the flowering plant family Moringaceae and is widely cultivated in Africa, Asia, and the Americas [3]. A large variety of nutritional and medicinal virtues have been shown in its leaves, seeds, flowers, and bark [4,5]. Its leaves are, for example, commonly consumed as vegetables and nutritional supplements; the seeds are taken fresh, dried or as roasted tea. The leaves and seeds of MO are rich in protein, lipids, vitamins, minerals, and phytochemicals. They have been used to treat and protect against various diseases including inflammation, carcinomas, hepatorenal and cardiovascular disorders, and DM [6,7].

Cumulative evidence has suggested the potentially beneficial roles of MO in glucose and lipid metabolism [8]. However, a limited number of clinical studies have been conducted on individuals with DM or glucose intolerance; moreover, these results have, as yet, been inconsistent. A single dose of MO in a meal significantly improved glucose tolerance [9] while chronic consumption of MO for 4 weeks did not alter fasting plasma glucose or hemoglobin A1C levels compared with that of placebo [10]. In healthy controls, no significant changes in glucose levels were reported [11], while a 3-month treatment with MO significantly decreased fasting glucose levels [12]. These inconsistencies are presumably due, in part, to the relatively limited number of recruited subjects and the differences in study design among clinical trials. Therefore, the aim of this study was to assess the effect of MO in the blood glucose and lipid levels of diabetic rodent models by meta-analyzing the currently available studies and attempting to sort out the potential source of heterogeneity that may lead to the discrepancies in the current literature with subgroup and meta-regression analyses.

## 2. Results

### 2.1. Search Results

**Figure 1:**
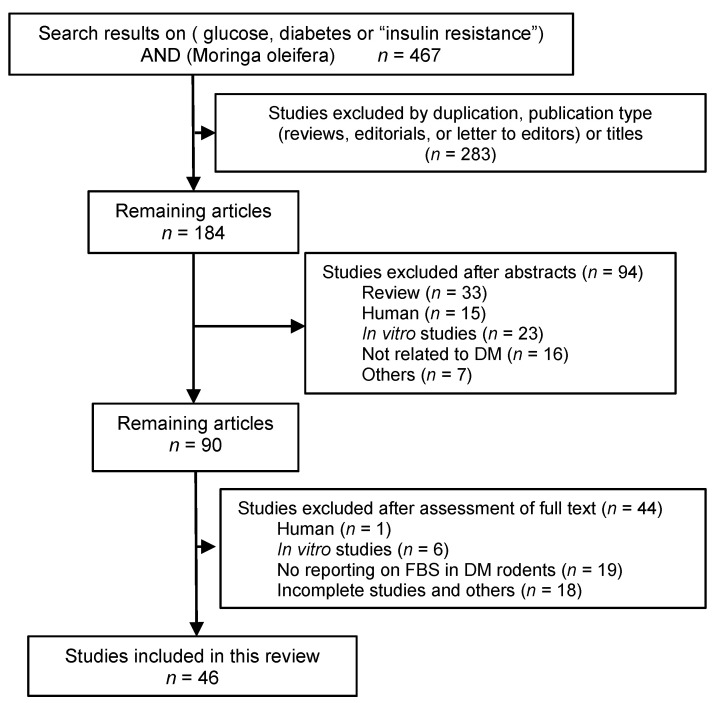
Flow diagram of literature search and selection process.

The flowchart of our literature search is shown in Figure 1. It resulted in a total of 467 articles (328 from Embase and 139 from PubMed). Upon removal of the duplicates and reviews of the titles and abstracts, 90 articles moved to a full-text assessment. The majority of these articles were excluded from the original meta-analysis because they failed to report blood glucose or lipid levels in DM rodent models treated with MO extracts. Therefore, 48 studies in 46 articles were finally included in the meta-analyses.

### 2.2. Study Characteristics and Quality Assessment

The main characteristics of each included study are summarized in Table 1. Studies have been published since 2003. The sample sizes ranged from 10 to 40 in each study. In the included studies, animals were treated with extracts isolated from leaves in 33 studies, seeds in 6 studies, and others, such as fruits and bark, in 9 studies. *db/db* mice were used in 2 studies, diet-induced obese diabetic rodents in 13 studies (4 in mice and 9 in rats) and dexamethasone (dexa)-induced insulin-resistant rats in 2 studies and chemical (alloxan or streptozotocin (STZ))-induced DM rodent models in 31 studies (30 in rats and 1 in mice). Six studies used plasma, 13 used serum, and 23 used whole blood samples for blood glucose measurement. Males were used in 33 studies, females in 7, and both in 7 studies. The detailed quality assessment of each study is shown in Table S1. The study quality was fair in general with the risk of bias judged to be low to medium.

### 2.3. MO Extracts Reduced Blood Glucose Levels in Some DM Rodent Models

#### 2.3.1. Forestplot Analysis

**Figure 2:**
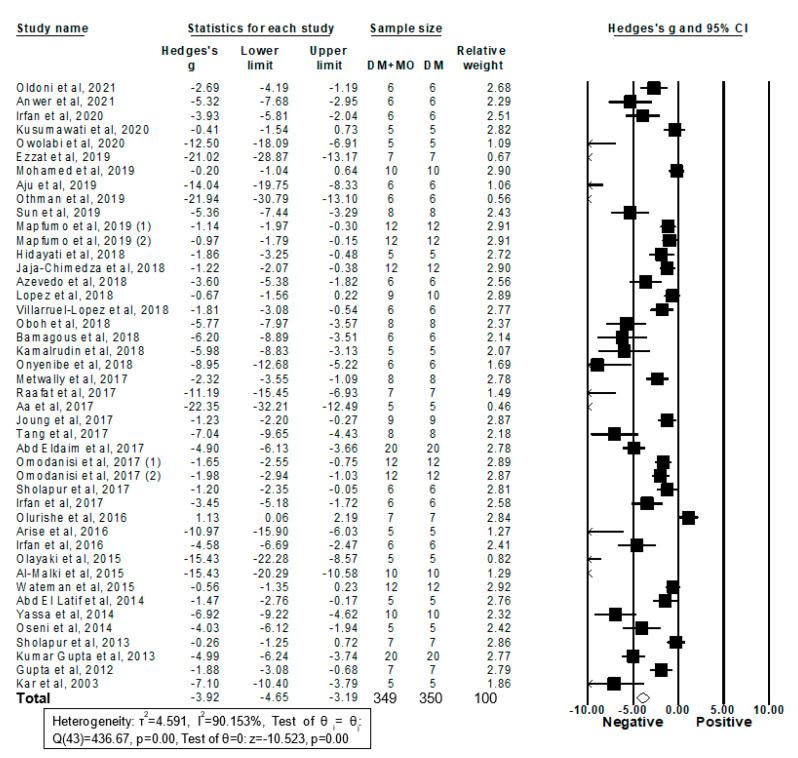
Meta-analysis of Hedges’ g of blood glucose levels in DM rodents with and without treatment with MO. Summary estimates were analyzed using a random-effects model. CI, confidence interval.

Forty four studies from 43 articles enrolling 349 diabetic rodents treated with MO extracts and 350 diabetic controls treated with vehicles reported their blood glucose levels and were included in this meta-analysis (Figure 2). Thirty-eight studies showed that treatment with MO extracts significantly reduced blood glucose levels in DM rodent models; six studies did not observe any significant effects. By pooling all those studies using a random-effects model, the results revealed that MO extracts reduce blood glucose levels in DM rodents (g = −3.92, 95% confidence interval (CI) −4.65 to −3.19; *I*^2^ = 90.15%, *p* = 0.00) and that the heterogeneity between studies was high (*I*^2^ ≥ 75%) (Figure 2). To determine the influence of each study on the overall result, the stability of the results was next evaluated using a leave-one-out strategy. Upon removal of each individual study, all the re-pooled summary estimates remained unchanged compared with the primary estimates with the effect sizes ranging from −4.05 (−4.81 to −3.30) to −3.72 (−4.43 to −3.01).

#### 2.3.2. Subgroup and Meta-Regression Analyses

To identify significant categorical covariates which explain the between-study heterogeneity in the meta-analysis, subgroup analyses were performed by DM models, sex, type of rodents and of blood samples and MO parts. MO administration significantly reduced blood glucose levels in chemical- and diet-induced DM rodents and *db/db* mice; however, it showed little effect in dexa-induced DM models. Moreover, an effect of MO administration on glucose levels may be different among males, females, and both (*p* = 0.049). MO parts (leaves, seeds, vs. others) (*p* = 0.82), types of rodent (rats vs. mice) (*p* = 0.75), and blood samples (plasma, serum, vs. whole blood) (*p* = 0.42) were not significant covariates (Table 2).

**Figure 3:**
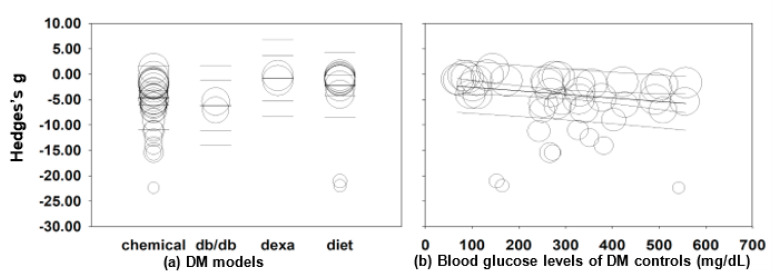
Meta-regression analyses for Hedges’ g of blood glucose levels and DM models (**a**) or blood glucose levels of DM controls (mg/dL) (**b**). Summary estimates were analyzed using a random-effects model.

Meta-regression analyses were next performed to identify additional continuous moderators which explain the between-study heterogeneity. The type of DM model was found to be a significant covariate to explain approximately 15% of between-study variance (*R*^2^ = 0.15); the average blood glucose levels of the vehicle group were also responsible for 11% of the overall heterogeneity (*R*^2^ = 0.11) (Figure 3). However, MO dose, the treatment period and the total MO dose administered during the study period did not affect the variance. Taken together, the high heterogeneity of the 44 pooling studies is, at least partially, due to the variety of rodent DM models and blood glucose levels of the vehicle-treated DM group.

#### 2.3.3. Subgroup and Meta-Regression Analyses in Chemical-Induced DM Rodents

MO administration significantly reduced blood glucose levels in chemical-induced DM rodents (Table 2). To examine significant covariates which influence the blood glucose levels of chemical-induced DM models (alloxan- and STZ-induced DM models combined), subgroup analyses were performed by sex, MO parts, and blood sample; none of them were found to be significant covariates (sex (*p* = 0.06), MO parts (*p* = 0.15), and blood sample type (*p* = 0.71)) (Table 3). The meta-regression analyses were also performed in chemical-induced DM models. MO dose, the treatment period, total MO dose administered during the study period or the average blood glucose levels of the vehicle group did not affect the variance. In sum, no additional significant covariates to type of DM rodent models found in the original meta-analysis were identified in chemical-induced DM rodents.

#### 2.3.4. Subgroup and Meta-Regression Analyses in Diet-Induced DM Rodents

MO treatment significantly reduced blood glucose levels in diet-induced DM rodents (Table 2). Subgroup and meta-regression analyses were then performed in diet-induced DM models. An effect of MO treatment on glucose levels may not be significantly different between males and females (*p* = 0.98), MO parts (*p* = 0.63), or type of blood sample (*p* = 0.10) (Table 3). MO dose, the treatment period, total MO dose administered during the study period, or the average blood glucose levels of the vehicle group did not affect the variance. Taken together, no significant covariates were found in diet-induced DM rodents.

#### 2.3.5. Assessment of Publication Bias

**Figure 4:**
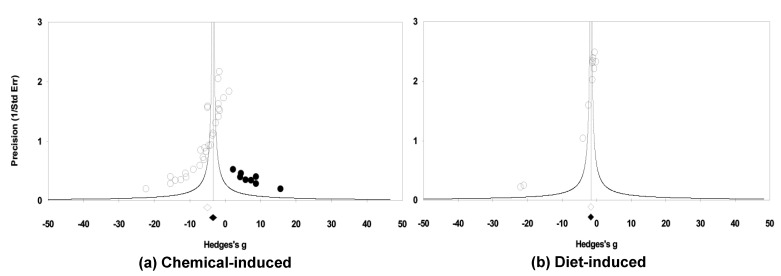
Funnel plots of standard error by Hedges’ g of blood glucose levels in chemical- (**a**) and diet-induced (**b**) DM rodents treated with or without MO. Open and closed diamonds indicate the imputed summary estimates before and after Duval and Tweedie’s Trim and Fill adjustment, respectively, in random-effects models. Closed circles represent imputed studies.

It was suggested that MO effects on blood glucose levels differ significantly among chemical and diet-induced DM models; therefore, publication bias was assessed separately by two DM models, chemical- and diet-induced, using the random-effects model. Significant evidence of publication bias was observed in the analyses of the effect of MO extracts on blood glucose levels as indicated by funnel plots (clear circles) in chemical-induced DM models. Duval and Tweedie’s Trim and Fill analysis found eight imputed studies (closed circles) whose adjusted g (95% CIs) was −3.43 (−4.52 to −2.35) (Figure 4a). No significant publication bias was detected in diet-induced DM models (Figure 4b). The number of studies for *db/db* and dexa-induced DM models was only two each; therefore, we did not conduct the analysis for publication bias. Taken together, publication bias existed in chemical-induced DM rodents; however, the findings that MO treatment had a positive impact on blood glucose levels in both chemical- and diet-induced DM rodents stand even after the trim and fill adjustment.

### 2.4. The Administration of MO Extracts Improved Diabetic Dyslipidemia in DM Rodent Models

#### 2.4.1. Triglyceride (TG) Levels

**Figure 5:**
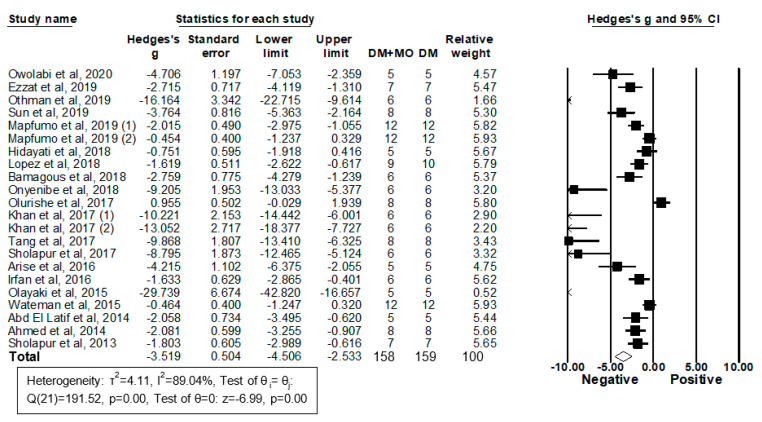
Meta-analysis of Hedges’ g of triglyceride levels in DM rodents with and without treatment with MO. Summary estimates were analyzed using a random-effects model. CI, confidence interval.

Twenty-two studies from 20 articles enrolling 158 diabetic rodents treated with MO extracts and 159 diabetic controls treated with vehicles reported serum or plasma TG levels and were included in the meta-analysis (Figure 5).

Eighteen studies showed that treatment with MO extracts significantly reduced TG levels in DM rodent models; four studies observed no significant effects. By pooling all those studies using a random-effects model, results revealed that MO extracts reduce blood TG levels (g = −3.52, 95% CI −4.51 to −2.53; *I*^2^ = 89.04%, *p* = 0.00) and that the heterogeneity between studies is high (Figure 5). To test the influence of each study on the overall result, the stability of results was next examined using a leave-one-out strategy. Upon removal of each individual study, all the re-pooled summary estimates remained unchanged compared with the primary estimates with the effect sizes ranging from −3.78 (−4.84 to −2.72) to −3.22 (−4.17 to −2.26).

Subgroup and meta-regression analyses were next performed by DM models, sex, MO parts, MO dose, the treatment period, and total MO dose administered during the study period. None of these factors were found to affect the variance. Publication bias was then assessed using the random-effects model followed by Duval and Tweedie’s Trim and Fill analysis. Significant evidence of publication bias was observed in the analysis of the MO effect on serum/plasma TG levels. Moreover, three imputed studies were found; the overall adjusted g was −2.99 (−4.03 to −1.95). Taken together, publication bias was observed in DM rodents; however, the findings that MO treatment had a positive impact on serum/plasma triglyceride levels in DM rodents seems to stand after the trim and fill adjustment.

#### 2.4.2. Total Cholesterol (TC) Levels

**Figure 6:**
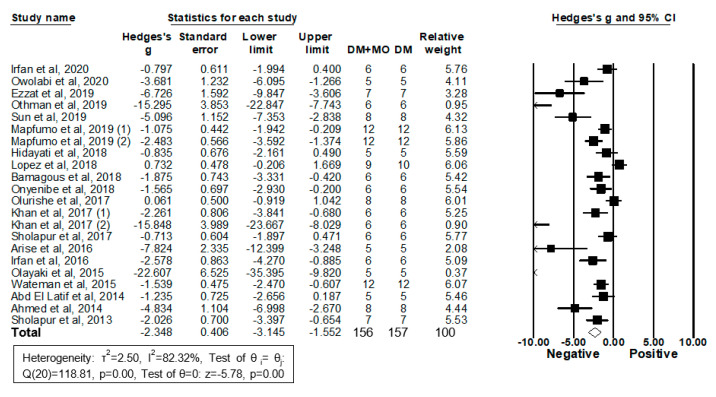
Meta-analysis of Hedges’ g of TC levels in DM rodents with and without treatment with MO. Summary estimates were analyzed using a random-effects model. CI, confidence interval.

Twenty-two studies from 20 articles enrolling 156 diabetic rodents treated with MO extracts and 157 diabetic controls treated with vehicles reported their serum/plasma TC levels and were included in the meta-analysis (Figure 6).

Twenty two studies showed that treatment with MO extracts significantly reduced TC levels in DM rodent models; five studies observe no significant effects. The analysis using a random-effects model showed that MO extracts reduce TC levels (g = −2.35, 95% CI −3.15 to −1.55; *I^2^* = 82.32%, *p* = 0.00) and that the heterogeneity between studies is relatively high (Figure 6). A leave-one-out strategy was used to determine the influence of each study on the overall result. After removing each individual study, all the re-pooled summary estimates remained unchanged in comparison to the primary estimates with the effect sizes ranging from −2.51 (−3.34 to −1.68) to −2.16 (−2.94 to −1.38).

Subgroup analyses by DM models, sex, and MO parts and meta-regression analyses by daily MO dose, the treatment period, and total MO dose administered did not find any factors responsible for the heterogeneity. Publication bias was assessed using the random-effects model followed by Duval and Tweedie’s Trim and Fill analysis. Significant evidence of publication bias was observed in the analysis of the MO effect on serum/plasma TC levels. Three imputed studies suggested the overall adjusted g −2.07 (−2.94 to −1.20). Taken together, MO treatment significantly reduces serum/plasma total cholesterol levels in DM rodents even after adjusting the small study effects caused by publication bias.

#### 2.4.3. High-Density Lipoprotein Cholesterol (HDL-C) Levels

**Figure 7:**
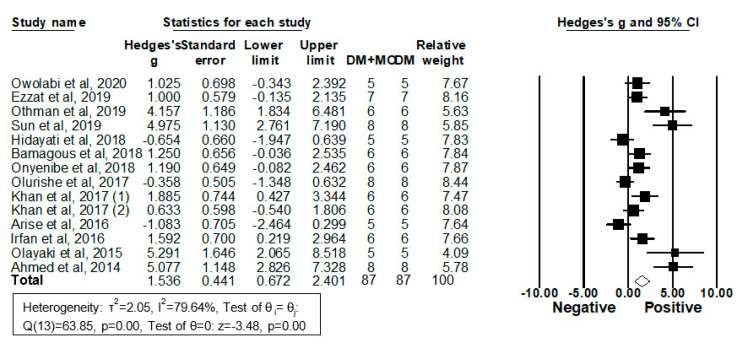
Meta-analysis of Hedges’ g of HDL-C levels in DM rodents with and without treatment with MO. Summary estimates were analyzed using a random-effects model. CI, confidence interval.

Fourteen studies from 13 articles enrolling 87 diabetic rodents treated with MO extracts and 87 treated with vehicles reported their serum or plasma HDL-C levels and were included in the meta-analysis (Figure 7).

By pooling all those studies using a random-effects model, results revealed that MO extracts increase HDL-C levels (g = 1.54, 95% CI 0.68 to 2.40; *I^2^* = 79.6%, *p* = 0.00) and that the heterogeneity between studies is relatively high (Figure 7). Upon removal of each individual study, all the re-pooled summary estimates varied compared with the primary estimates with the effect sizes ranging from −1.08 (−2.46 to 0.30) to 5.29 (2.07 to 8.52).

Subgroup analyses were performed by DM models, sex, and MO parts. MO treatment significantly increased HDL-C levels in diet-induced DM rodents but not in chemical-induced DM models (Table 4). Sex and MO parts were not significant covariates.

Univariate meta-regression analyses were performed next. Type of DM models was found to be a significant covariate to explain approximately 21% of between-study variance (*R*^2^ = 0.21). Moreover, the treatment period, MO dose, and total MO dose administered during the study period were 29, 2, and 24%, respectively, responsible for the variance. Taken together, the high heterogeneity of the 14 pooling studies was apparently, at least, in part due to the variety of rodent DM models, MO dose, duration, and the total administered MO dose.

To further examine significant covariates which influence HDL-C levels in diet-induced DM rodents, subgroup analyses were performed by sex and MO parts. Neither factor exhibited the significant differences between the groups treated with and without MO (sex; *p* = 0.93. MO parts; *p* = 0.06). The experimental period and total MO doses administered during the period were found to be significant covariates, which explain 91 and 68% of the between study-variance, respectively, in diet-induced DM rodents in meta-regression analyses. Publication bias was then assessed using the random-effects model followed by Duval and Tweedie’s Trim and Fill analysis in diet-induced DM animals. Significant evidence of publication bias was observed in the analysis of the MO effect on serum/plasma HDL-C levels; one imputed study eliminated the MO’s significant effect on HDL-C levels (adjusted g 1.71 (−0.19 to 3.61)). Thus, MO administration apparently increases HDL-C levels of diet-induced DM models dependently on the treatment period and MO doses; however, this finding is likely due to publication bias. In sum, MO treatment does not affect HDL-C levels in DM rodents.

Few clinical trials have been conducted to elucidate an effect of MO treatment on lipid profiles in patients with DM. Kumari et al. reported that diabetic patients treated with MO at a daily dose of 8 g for 40 days show significantly reduced serum TG and TC levels without altering HDL-C levels [58]; our meta-analyses on diabetic dyslipidemia in DM rodent models supports the result of the clinical study. Moreover, it might further suggest a possibility that MO administration could play a critical role in normalizing glucose and lipid profiles and consequently delaying the onset and progress of serious cardiovascular diseases in patients with DM.

## 3. Discussion

### 3.1. Main Findings

This study would be the first meta-analysis that summarizes the evidence that MO treatment reduces blood glucose, serum/plasma triglyceride, and the total cholesterol levels of DM rodent models. We showed that elevated blood glucose levels of DM rodents are significantly reduced by daily oral gavage of MO extracts compared with those of DM controls. This association was, at least partially, influenced by the type of DM models and the glucose levels of the DM control group. No significant covariates were found, which influenced the effects of MO on DM-mediated hypertriglyceridemia and hypercholesterolemia. Publication bias was found in every meta-analysis, which suggests that manuscripts which suggest no beneficial effects of MO on those parameters might have been less likely to be published in DM rodent models.

### 3.2. Interpretation

#### 3.2.1. Nutritional Characteristics of MO

Various parts of MO, and leaves in particular, have played a critical role as a source of essential nutrients and medicine for undernourished individuals. USDA FoodData Central has shown that 100 g of raw leaves, for example, containing carbohydrate (≈8.28 g), protein (≈9.4 g), lipid (≈1.4 g), vitamins (A equiv., ≈378 μg; various Bs; C, ≈51.7 mg; folate, ≈40 μg), minerals (potassium, ≈337 mg; calcium, ≈185 mg; magnesium, ≈42 mg; iron, ≈4 mg), and dietary fiber (≈2 g) [59]. The analysis of dried MO demonstrated that leaves are a rich source of omega-3 and omega-6 polyunsaturated fatty acids and various phytochemicals [58,60]. The relatively wide range of variability in nutritional data was also reported, mainly due to genetic background, soil, climate, season, and plant; the use of different procedures of processing and storage and analytical techniques may also increase the variations [61,62,63,64].

#### 3.2.2. Pharmacological Properties of MO Extracts

**Figure 8:**
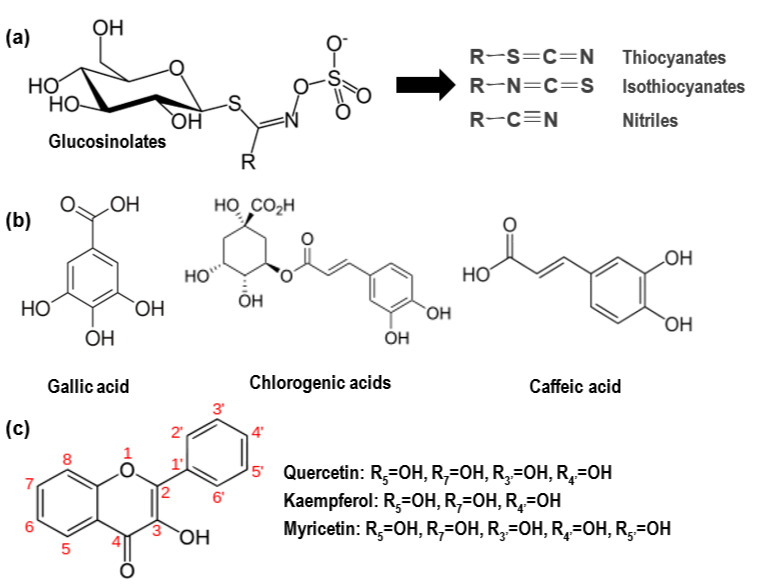
Structures of major phytochemicals of MO leaf extracts. (**a**) glucosinolates, (**b**) phenolic acids, and (**c**) flavonoids.

MO presents a wide variety of biological activities. The leaf is the most commonly used plant part for therapeutic purposes and its main phytochemicals include glucosides (glucosinolates), phenolics (phenolic acids and flavonoids), and carotenoids (β-carotene) [18,29,65,66]. The structures of different classes of major phytochemicals present in the MO leave extracts are shown in Figure 8 [67,68].

Glucosinolates are bioactive after being hydrolyzed by the endogenous enzyme myrosinase to thiocyanates, isothiocyanates, and nitriles, which are the active molecules with hypoglycemic effects [49,69,70]. Antioxidant activity can protect organs such as the pancreas, liver, and adipose tissues from hyperglycemia-mediated oxidative stress [38,51,71,72,73]. This capacity is attributed to a high concentration of polyphenolics, including phenolic acids (gallic acid, chlorogenic acid, and caffeic acid) and flavonoids (quercetin, kaempferol, and myricetin) [44,64,74].

Several anti-diabetic pharmacological mechanisms have been suggested in MO extracts. First, pancreatic α-amylase catalyzes the breakdown of polysaccharides into disaccharides and oligosaccharides [75]. Second, α-glucosidase is a carbohydrate-hydrolase which acts on the terminal α (1 → 4) bonds of starch and disaccharides to release α-glucose in the brush border of the small intestine. Several mono-glucosides of quercetin and kaempferol in MO extracts were shown to have strong binding abilities to α-amylase and α-glucosidase; this suggests the potential capacities of the glucosides to inhibit these enzymatic activities [76,77]. The inhibitory effects of gallic acid on α-amylase and α-glucosidase activities are approximately 50% of those of a potent α-glucosidase inhibitor, acarbose [78]. Both chlorogenic and caffeic acids also demonstrate the inhibition of both enzymes (caffeic acid > chlorogenic acid) [79]. Third, sodium-glucose linked transporter 1 (SGLT1) in the mucosa of the small intestine facilitates the transport of D-glucose from the brush-border membrane of the small intestine into cells [80]. The flavonoids, quercetin and kaempferol, and the phenolic acid, chlorogenic acid, are reported to act as competitive inhibitors of the SGLT1 in the small intestine and reduce the absorption of glucose from the intestine [81,82,83]. Therefore, the inhibition of these three enzymatic activities could improve postprandial hyperglycemia in diabetic subjects. Fourth, hexokinase (HK) is an enzyme which phosphorylates glucose to yield glucose 6-phosphate in the first step of glycolysis and glucose 6-phosphate dehydrogenase (G6PD) participates in the pentose phosphate pathway, a metabolic pathway which supplies nicotinamide adenine dinucleotide phosphate and pentose [84,85]. MO is shown to restore the activities of those two glucose metabolizing enzymes and facilitate glycolysis and glucose utilization by the pentose phosphate pathway [32]. Fifth, MO contains dietary fibers in a range of 20~28%, suggesting that those fibers might reduce glucose absorption from the intestine [58,61,86]. Finally, it is also reported that MO increases the expression of insulin receptor and insulin receptor substrate 1 in the liver and glucose transporter 4 in the liver and muscles, to increase insulin sensitivity and glucose uptake into cells, respectively [19,24].

Increased lipid and protein peroxidation and mRNA expression of fatty acid synthase (FAS) and 3-hydroxy-3-methylglutaryl-coenzyme A (HMG-CoA) reductase in the liver have been reported in DM models [13,18,21,30,32]. Those modifications may play a role in the development of diabetic dyslipidemia. FAS is an enzyme which mainly catalyzes fatty acid synthesis of palmitate from acetyl-CoA and malonyl-CoA [87]. HMG-CoA reductase is the rate-limiting enzyme which catalyzes the conversion of HMG-CoA to mevalonic acid, an essential step in the biosynthesis of cholesterol [88,89]. MO administration reduces oxidative stress and fatty acid and cholesterol synthesis, resulting in the normalization of lipid profiles [13,18,21,30,32]. Moreover, in adipose tissues, MO was shown to normalize increased mRNA levels of leptin and resistin, and decreased those of adiponectin, melanocortin receptor-4, and peroxisome proliferator-activated receptors α and γ; those changes could also lead to the normalization of body composition and insulin sensitivity of DM rodents [14,18,33].

#### 3.2.3. Strength and Limitations

The primary strength of this meta-analysis is the inclusion of a relatively large number of DM mouse and rat models and a focus on the effect of MO on their blood glucose, TG, TC, and HDL-C levels. We also systematically assessed various cofounding factors and publication biases. This meta-analysis also has several limitations. First, although a broad literature search was applied using two electronic databases, the number of articles assessing an effect of MO in *db/db* mice and dexa-induced diabetic models was quite small and the language restriction and the exclusion of ambiguous literature might further increase the risk of publication bias. Second, substantial evidence of heterogeneity existed in our meta-analyses and this could not be fully explained by the pre-specified variables. This heterogeneity may potentially weaken the robustness of our findings. One of the possible reasons for the unexplained heterogeneity could be the relatively wide range of variability in the nutritional content of MO extracts; the nutritional data uniquely vary depending on the genetic background, soil, climate, season, and the use of different procedures of processing and storage [61,62,63,64]. Third, the included studies mostly used male rodents and the outcome could be different when more studies include females or both sexes. Fourth, dexa-induced insulin-resistant rodent models were generated by injection simultaneously during dietary modulation with or without MO in two included studies; thus, there is at least a slight possibility that the investigators’ technical skill for chemical injection could directly or indirectly have influenced the experimental outcome of blood glucose levels [43,54].

## 4. Materials and Methods

### 4.1. Data Sources and Search Strategies

A comprehensive literature search of electronic data bases, PubMed and Embase, was conducted using text words of (“*Moringa oleifera*” and (glucose, diabetes or “insulin resistance”)) up to 30 April 2021. In addition, the reference lists of the retrieved articles were manually searched to ensure that no relevant articles had been missed.

### 4.2. Inclusion and Exclusion Criteria

Peer-reviewed articles in the English language were eligible for inclusion when they fulfilled the following inclusion criteria: (i) studies used diabetic rodent models with or without treatment with MO for longer than 3 days; (ii) they also reported the blood glucose, triglyceride, total cholesterol, or high-density lipoprotein cholesterol levels of the DM rodents at the end of the treatment period. Studies were excluded if they were reviews, commentaries, editorials, letters, conference abstracts, duplicates, were not in English, or were not studied with the treatment for a period shorter than 3 days in rodent DM models. Unpublished research was not sought. Some authors were contacted by email to collect additional information. This meta-analysis was strictly conducted according to the PRISMA guidelines (Table S2).

### 4.3. Data Extraction and Quality Assessment

Titles and abstracts of the retrieved publications were screened initially for potentially eligible studies, which were subsequently evaluated by a full-text review. Data were collected by 3 authors (S.W., H.O., and M.S.) in an independent manner using a pre-designed standardized data extraction form, which includes dose, method, and period of administration, blood glucose and lipid (TG, TC, and HDL-C) levels, baseline age, sex, type of rodent DM models and their relevant controls.

Study quality was assessed by the Cochrane Collaboration “Risk of Bias” Tool [90]. The risk of bias for each quality variable in each criterion was assessed by 2 authors in an independent manner, and were judged as “low”, “unclear”, “high”, or “not applicable (N/A)” based on its description in each included study. Any disagreements in any phase were resolved by discussion until a consensus was achieved.

### 4.4. Data Synthesis and Analysis

Continuous variables were presented as means ± standard deviation (SD). For studies reporting the standard errors of means (SEs), the corresponding SDs were calculated by multiplying by the square root of the respective sample size. For studies providing glucose levels in mmol/L, these levels were converted into mg/dL using the conversion table offered by Joslin Diabetic Center at http://www.joslin.org/info/conversion_table_for_blood_glucose_monitoring.html (accessed on 5 January 2021). In studies providing serum/plasma TG, TC, and HDL-C levels in mmol/L, these levels were converted into mg/dL using the omni calculator offered at https://www.omnicalculator.com/health/cholesterol-units (accessed on 5 January 2021). For those studies with more than 1 measure of blood glucose, serum/plasma TG, TC, and HDL-C levels, the levels after the longest period of treatment with MO extracts, and at the treatment dose which gave the most robust difference in blood glucose levels between the two DM rodent groups were selected and included in the primary meta-analyses.

A standardized mean difference (Hedges’ g) transformation was used to calculate the related statistics, including variance and 95% CIs of each study and the summary effect size generated in the meta-analyses and publication bias assessment. The random-effects model was chosen in this study because it is more conservative and incorporates better between-study variability. Heterogeneity was assessed using *I^2^* statistics with its value ≥ 75% interpreted as evidence of substantial heterogeneity.

In some subgroup analyses, STZ- and alloxan-induced DM models are combined as chemical-induced models because both chemicals are used to induce DM by destructing pancreatic β cells in animals [91]. Subgroup and meta-regression analyses were performed based on the types of DM models (chemical-, dexa-, or diet-induced and *db/db* mice), MO parts, blood samples, and rodents (mice vs. rats), sex, MO dose, treatment period, total MO dose administered during the experimental period, blood glucose levels of DM control rodents to examine their influence to the outcome estimates. Sensitivity analyses were used to evaluate the robustness of the outcome estimates mainly by removing one study at a time with a repeat of the primary meta-analyses. Publication bias was assessed by funnel plots with Duval and Tweedie’s Trim and Fill analysis (random-effects model). All the statistical analyses were carried out using Comprehensive Meta-Analysis 3.0 (Biostat Inc., Englewood, NJ, USA) and STATA16 (StataCorp, College Station, TX, USA) software.

## 5. Conclusions

The present meta-analyses demonstrated that blood glucose, TG, and TC levels were significantly reduced in diabetic rodent models treated with MO extracts. The outcome of animal studies might not be immediately translated into the human condition because of the biophysiological diversity between species. However, our analyses could shed light on a future more practical use of the MO for the prevention and treatment of DM and its associated dyslipidemia in humans. Finally, it could have a profound impact on an increasing number of pre-diabetic patients worldwide, in particular, if herbal extracts such as MO could be developed as natural nutraceuticals for prevention, delayed onset, or progress of DM.

## Figures and Tables

**Table 1:** Characteristics of included studies in the meta-analysis.

Authors (Year)	Parts	Dose (mg/kg BW)	Duration Exposed to MO	Animal Models	Sex	*n* (T/no-T)	Age or Weight at the Baseline	Diet	Blood Sample for BS	Measurements
Oldoni et al. (2021) [13]	leaves	500	45 days	rats, STZ 55 mg/kg	males	6/6	90 days, 200–250 g	control	NS	BS
Anwer et al. (2021) [14]	leaves	400	21 days	rats, STZ 40 mg/kg	males	6/6	100–120 g	HFD	serum	BS
Irfan et al. (2020) [15]	leaves	1000	30 days	rats	males	6/6	6 wks old	HFD, 20% fructose in water	whole blood	BS, TC
Kusumawati et al. (2020) [16]	seeds	300	2 weeks	rats, alloxan 150 mg/kg	females	5/5	NS	control	whole blood	BS
Owolabi et al. (2020) [17]	pods	200	3 weeks	rats, alloxan 100 mg/kg	both	5/5	130–150 g	control	plasma	BS, TG, TC, HDL-C
Ezzat et al. (2020) [18]	leaves	400	1 month	rats	males	7/7	100 ± 20 g	HFD	serum	BS, TG, TC, HDL-C
Mohamed et al. (2019) [19]	leaves	300	4 weeks	rats	males	10/10	140–320 g	high fructose diet	serum	BS
Aju et al. (2019) [20]	leaves	300	60 days	rats, STZ 30 mg/kg	males	6/6	170–180 g	high energy diet	serum	BS
Othman et al. (2019) [21]	leaves	300	6 weeks	rats	males	6/6	200–230 g	HFD	serum	BS, TG, TC, HDL-C
Sun et al. (2019) [22]	leaves	120	8 weeks	*db/db* mice	males	8/8	6 wks old	control	serum	BS, TG, TC, HDL-C
Mapfumo et al. (2019) (1) (2) [23]	seeds	500	12 weeks	rats	females/males	12/12	3 wks old	high fructose diet	whole blood	BS, TG, TC
Hidayati et al. (2018) [24]	leaves	50	10 days	rats, STZ 65 mg/kg	males	5/5	NS	control	NS	BS, TG, TC, HDL-C
Jaja-Chimedza et al. (2018) [25]	seeds	0.54% in diet	12 weeks	mice	males	12/12	5 wks old	HFD	whole blood	BS
Azevedo et al. (2018) [26]	leaves	100	10 days	rats, STZ 45 mg/kg,	NS	6/6	280 ± 23 g	control	whole blood	BS
López et al. (2018) [27]	leaves	700	3 weeks	rats	males	9/10	250 ± 50 g	HFD, 10% fructose in water	whole blood	BS, TG, TC
Villarruel-Lopez et al. (2018) [28]	leaves	50	8 weeks	rats, alloxan 150 mg/kg	males	6/6	180–200 g	control	whole blood	BS
Oboh et al. (2018) [29]	leaves	4% in diet	14 days	rats, STZ 60 mg/kg	males	8/8	260–280 g	control	whole blood	BS
Bamagous et al. (2018) [30]	leaves	200	30 days	rats, STZ 55 mg/kg	males	6/6	8–12 wks old, 200–250 g	control	whole blood	BS, TG, TC, HDL-C
Kamalrudin et al. (2018) [31]	fruits	500	21 days	rats, STZ 50 mg/kg	males	5/5	10–12 wks old, 300–500 g	control	whole blood	BS
Onyenibe et al. (2018) [32]	leaves	400	30 days	rats, STZ 60 mg/kg	males	6/6	2 wks old, 120–140 g	control	whole blood	BS, TG, TC, HDL-C
Metwally et al. (2017) [33]	aerial parts	600	12 weeks	rats	females	8/8	13 wks old, 130 ± 10 g	high cholesterol diet	serum	BS
Olurische et al. (2017) [34]	leaves	300	6 weeks	rats, alloxan 150 mg/kg	both	8/8	NS	control	NA	TG, TC, HDL-C
Raafat et al. (2017) [35]	seeds	40–80	8 days	mice, alloxan 180 mg/kg	males	7/7	18–29 g	control	whole blood	BS
Aa et al. (2017) [36]	leaves	400	24 days	rats, alloxan 150 mg/kg	both	5/5	12 wks old, 100 g	control	whole blood	BS
Khan et al. (2017) (1) (2) [37]	leaves	100	3 weeks	rats, STZ 45 mg/kg	females	6/6	200–250 g	control	NA	TG, TC, HDL-C
		200		mice		6/6	6 wks old	HFD	NA	
Joung et al. (2017) [38]	leaves	250	10 weeks	mice	males	9/9	4 wks old	HFD	whole blood	BS
Tang et al. (2017) [39]	leaves	150	5 weeks	*db/db* mice	males	8/8	7 wks old	control	plasma	BS, TG
Abd Eldaim et al. (2017) [40]	leaves	250	18 days	rats, alloxan 150 mg/kg	both	20/20	110–170 g	control	plasma	BS
Omodanisi et al. (2017) (1) [41]	leaves	250	6 weeks	rats, STZ 55 mg/kg	males	12/12	200–250 g	control	serum	BS
Omodanisi et al. (2017) (2) [42]	leaves	250	6 weeks	rats, STZ 55 mg/kg	males	12/12	10 wks old, 200–250 g	control	plasma	BS
Sholapur et al. (2017) [43]	stem bark	140	11 days	rats, dexa 1 mg/kg	males	6/6	180–200 g	control	serum	BS, TG, TC
Irfan et al. (2017) [44]	leaves	1000	14 days	rats, STZ 45 mg/kg	males	6/6	8–9 wks old, 230 ± 30 g	control	whole blood	BS
Olurishe et al. (2016) [34]	leaves	300	6 weeks	rats, alloxan 150 mg/kg	both	8/8	NS	control	whole blood	BS
Arise et al. (2016) [45]	flowers	300	21 days	rats, STZ 45 mg/kg	males	5/5	151 ± 5 g	control	whole blood	BS, TG, TC, HDL-C
Irfan et al. (2016) [46]	leaves	500	14 days	rats, STZ 45 mg/kg	males	6/6	5–6 wks old, 170–200 g	control	whole blood	BS, TG, TC, HDL-C
Olayaki et al. (2015) [47]	leaves	600	6 weeks	rats, alloxan 120 mg/kg	males	5/5	6 wks, 150–180 g	control	whole blood	BS, TG, TC, HDL-C
Al-Malki et al. (2015) [48]	seeds	100	4 weeks	rats, STZ 60 mg/kg	males	10/10	180–200 g	control	serum	BS
Waterman et al. (2015) [49]	leaves	5% extracts in diet	12 weeks	mice	males	12/12	5 wks old	HFD	whole blood	BS, TG, TC
Abd El Latif et al. (2014) [50]	leaves	250	18 days	rats, alloxan 100 mg/kg	females	5/5	130–170 g	control	serum	BS, TG, TC
Yassa et al. (2014) [51]	leaves	200	8 weeks	rats, STZ 60 mg/kg	males	10/10	12 months old, 180–200 g	control	plasma	BS
Ahmed et al. (2014) [52]	aerial parts	600	12 weeks	rats	female	8/8	13 wks old, 130 ± 10 g	high cholesterol diet	NA	TG, TC, HDL-C
Oseni et al. (2014) [53]	leaves	30% in 0.5 mL water	1 week	rats, alloxan 35 mg/kg	males	5/5	74.6–87.3 g	control	whole blood	BS
Sholapur et al. (2013) [54]	stem bark	250	11 days	rats, dexa 1 mg/kg	males	7/7	180–200 g	control	plasma	BS, TG, TC
Kumar Gupta et al. (2013) [55]	leaves	100	24 weeks	rats, STZ 45 mg/kg	both	20/20	200–250 g	control	whole blood	BS
Gupta et al. (2012) [56]	pods	300	21 days	rats, STZ 50 mg/kg	both	7/7	170–230 g	control	serum	BS
Kar et al. (2003) [57]	stem bark	250	1 week	rats, alloxan 100 mg/kg	males	5/5	150–200 g	control	serum	BS

STZ: streptozotocin, HFD: high fat diet, NA: not applicable, NS: not specified, T/no-T: treatment/no treatment with MO, wks: weeks, BS: blood sugar, TG: triglyceride, TC: total cholesterol, HDL-C: high-density lipoprotein cholesterol.

**Table 2:** Subgroup analyses of blood glucose levels.

Subgroups	Effect Size					Heterogeneity (*I*^2^)	Test of Group Difference (*p*)
	No. of Studies	g	95% CI		*p*-Value		
**DM rodent models**							
Chemical	29	−4.65	−5.51	−3.78	<0.001	89.80	0.00
*db/db*	2	−6.15	−9.35	−2.95	<0.001	<0.001	
Dexa	2	−0.73	−3.57	2.12	0.62	32.13	
Diet	11	−2.13	−3.44	−0.82	0.001	84.82	
**Sex**							
Male	33	−4.33	−5.21	−3.46	<0.001	89.84	0.05
Females	4	−1.33	−3.58	0.92	0.25	42.56	
Both	6	−4.27	−6.37	−2.16	<0.001	95.15	
**Parts**							
Leaves	30	−4.09	−5.00	−3.18	<0.001	90.69	0.82
Seeds	6	−3.38	−5.36	−1.39	0.001	91.12	
Others	8	−3.98	−5.77	−2.19	<0.001	87.62	
**Rodent type**							
mice	6	−3.66	−5.60	−1.72	<0.001	91.18	0.75
rats	38	−4.01	−4.81	−3.20	<0.001	90.66	
**Blood sample**							
Plasma	6	−4.67	−6.71	−2.63	<0.001	93.17	0.42
Serum	13	−4.71	−6.18	−3.24	<0.001	91.18	
Whole blood	23	−3.64	−4.69	−2.59	<0.001	89.82	

**Table 3:** Subgroup analyses of blood glucose levels in chemical- and diet-induced DM rodent models.

DM Type	Subgroups	Effect Size					Heterogeneity (*I*^2^)	Test of Group Difference (*p*)
		No. of Studies	g	95% CI		*p*-Value		
Chemical-induced	**Sex**							
	Males	20	−5.78	−7.12	−4.44	<0.001	86.71	0.06
	Females	2	−0.93	−4.74	2.87	0.63	31.22	
	Both	6	−4.64	−7.11	−2.16	<0.001	95.15	
	**Parts**							
	Leaves	21	−4.46	−5.74	−3.17	<0.001	89.79	0.15
	Seeds	3	−7.40	−11.06	−3.74	<0.001	96.35	
	Others	5	−6.76	−9.61	−3.90	<0.001	87.93	
	**Blood sample**							
	Plasma	4	−5.74	−8.88	−2.60	<0.001	90.90	0.71
	Serum	6	−6.37	−8.98	−3.66	<0.001	91.36	
	Whole blood	17	−5.05	−6.60	−3.49	<0.001	90.87	
Diet-induced	**Sex**							
	Males	9	−1.73	−2.77	−0.68	0.001	86.98	0.98
	Females	2	−1.70	−3.68	0.28	0.09	59.05	
	**Parts**							
	Leaves	7	−2.04	−3.32	−0.76	0.002	90.16	0.63
	Seeds	3	−1.11	−2.75	0.53	0.18	0.00	
	Others	1	−2.32	−5.30	0.65	0.13	0.00	
	**Blood sample**							
	Serum	4	−3.15	−5.06	−1.24	0.001	94.47	0.10
	Whole blood	7	−1.29	−2.36	−0.25	0.02	47.90	

**Table 4:** Subgroup analyses of HDL-C levels.

Subgroups	Effect Size					Heterogeneity (*I*^2^)	Test of Group Difference (*p*)
	No. of Studies	g	95% CI		*p*-Value		
**DM rodent models**							
Chemical	9	0.85	−0.11	1.82	0.084	71.63	0.03
*db/db*	1	4.98	1.64	8.31	0.003	0.00	
Diet	4	2.33	0.82	3.84	0.002	82.86	
**Sex**							
Males	9	1.63	0.48	2.78	0.005	81.02	0.42
Females	3	2.30	0.33	4.26	0.022	83.18	
Both	2	0.30	−1.97	2.58	0.794	61.19	
**MO parts**							
Leaves	11	1.60	0.59	2.61	0.00	76.47	ND
Others	3	1.39	−0.56	3.34	0.16	90.53	

## Supplementary Materials

The following are available online. Table S1: Risk of Bias; Table S2: PRISMA checklist.
